# A nomogram model to predict the risk of drug-induced liver injury in patients receiving anti-tuberculosis treatment

**DOI:** 10.3389/fphar.2023.1153815

**Published:** 2023-05-18

**Authors:** Songjun Ji, Bin Lu, Xinling Pan

**Affiliations:** ^1^ Department of Biomedical Sciences Laboratory, Affiliated Dongyang Hospital of Wenzhou Medical University, Dongyang, China; ^2^ Department of Infectious Diseases, Affiliated Dongyang Hospital of Wenzhou Medical University, Dongyang, China

**Keywords:** nomogram, drug-induced liver injury, anti-tuberculosis treatment, prediction model, risk factors

## Abstract

**Objectives:** To establish an individualized nomogram to predict the probability of drug-induced liver injury (DILI) in tuberculosis patients receiving anti-tuberculosis treatment.

**Methods:** The clinical information of patients admitted to a tertiary hospital between January 2010 and December 2022 was retrospectively reviewed from the clinical records. Patients with baseline liver diseases (hepatis B or C infection and fatty liver) or taking liver protective drugs were excluded. The maximum values in liver function test within 180 days after anti-tuberculosis treatment were collected to determine the occurrence of DILI. The candidate variables used for establishing prediction model in this study were the last results within the 30 days before the treatment onset. The final variables were included after univariate and multivariate logistic regression analyses and applied to establish the nomogram model. The discrimination power and prediction accuracy of the prediction model were assessed using the area under the receiver operating characteristic (AUC) curve and a calibration chart. The clinical effectiveness was assessed via decision curve analysis (DCA). The established model was validated in two validation groups.

**Results:** A total of 1979 patients with 25 variables were enrolled in this study, and the incidence of DILI was 4.2% (n = 83). The patients with complete variables were divided into training group (n = 1,121), validation group I (n = 492) and validation group II (n = 264). Five variables were independent factors for DILI and included in the final prediction model presented as nomogram: age (odds ratio [OR] 1.022, *p* = 0.023), total bilirubin ≥17.1 μmol/L (OR 11.714, *p* < 0.001), uric acid (OR 0.977, *p* = 0.047), neutrophil count (OR 2.145, 0.013) and alcohol consumption (OR 3.209, *p* = 0.002). The AUCs of the prediction model in the training group, validation group I and validation group II were 0.833, 0.668, and 0.753, respectively. The *p*-values of calibration charts in the three groups were 0.800, 0.996, and 0.853. The DCA curves of the prediction model were above the two extreme curves.

**Conclusion:** The nomogram model in this study could effectively predict the DILI risk among patients under anti-tuberculosis drug treatment.

## Introduction

*Mycobacterium tuberculosis* (MTB) is the pathogen that causes tuberculosis (TB) in humans and constitutes a great threat to global public health. The World Health Organization (WHO) reported that approximately 10.6 million people fell ill due to TB in 2021 worldwide, and this number is 4.4% higher than that in 2020 (Global tuberculosis report 2021, 2021; Global tuberculosis report 2022, 2022). Although the success rates of TB treatments can be as high as 86%, TB-caused mortality ranked second after COVID-19–caused mortality among infection-caused deaths (Global tuberculosis report 2022, 2022). Once the diagnosis of TB is confirmed based on clinical manifestations, radiological examination, and laboratory tests, a long course of antibiotic treatment is needed to control the growth of MTB. However, combined drug treatment is often accompanied by adverse effects, among which drug-induced liver injury (DILI) and gastrointestinal syndromes are the two most common events (Zhang et al., 2015; El Hamdouni et al., 2020; Wang et al., 2022a). Adverse effects frequently cause patients to abort the treatment, which might cause treatment failure, induced drug resistance, and transmission of the infection. Thus, it is essential to identify biomarkers for predicting DILI in patients with TB.

**S**everal studies on the possible risk factors for DILI in patients with TB have suggested complex mechanisms underlying DILI (Medina-Caliz et al., 2016; Ashby et al., 2021). In fact, combinatorial drug regimens containing various antibiotics are always prescribed to patients with TB, but the potential association between DILI and such combinatorial regimens has not been explored (Sterling et al., 2011; Ahmad et al., 2018; Lan et al., 2020; Xu et al., 2022). Moreover, the hepatic viral infections, patients’ age, nutritional status, and alcohol-consumption level of patients, as well as their clinical history of liver disease may affect liver function (Medina-Caliz et al., 2016; Sun et al., 2016; Chang et al., 2018; Jiang et al., 2021) and thereby contribute to DILI occurrence (Yew et al., 2018). In clinical practice, the patients with high risk of DILI based on the clinical experiences have been suggested for taking drugs for prophylaxis, resulting into the fact that patients who are not under liver protection are largely ignored (Saito et al., 2016; Xu et al., 2017). Novel urinary (Wang et al., 2022b) or blood (Ho et al., 2021; Wang et al., 2022c; Deng et al., 2022) biomarkers identified via high-throughput methods involving mass spectrometry or sequencing might indicate for potential role in predicting DILI, but they are far away from clinical practice due to uncertainty and high cost.

Routine laboratory examinations used in TB diagnosis may provide us with objective and quantitative parameters that can be screened for risk factors related to DILI. Prediction model can facilitate the screening for the significant risk factors in the real world, which has been found essential in diagnosis, treatment, and prognosis. Although several risk factors have been found associated with DILI, there are limited reports on the application of nomogram to predict DILI-associated risks in a large sample size (Ashby et al., 2021; Raj Mani et al., 2021; Zhong et al., 2021; Zhao et al., 2022). Here, a cohort of TB patients spanning 13 years were retrospectively reviewed, and a nomogram prediction model was established to evaluate the DILI risk following anti-TB treatment among patients who were negative for baseline liver diseases and liver protective drugs.

## Materials and methods

### Participants inclusion and exclusion

TB patients who met the inclusion criteria were enrolled via data extraction from clinical record mining database (supported by Le 9 Co., Ltd.): the patients who were diagnosed with tuberculosis infection and started their anti-TB drug treatment between January 2010 and December 2022. The database was constructed based on the clinical records from patients who were admitted to Affiliated Dongyang Hospital of Wenzhou Medical University. Exclusion criteria: the patients who took liver protective drugs for treatment or prophylaxis based on the drug usage record; the patients who were accompanied by HIV infection and liver cancer. The baseline liver diseases including hepatitis caused by HBV or HCV (by immunological test) and fatty liver (by radiological examination) were determined based on the extracted information from clinical record database.

### Definition

The definition criteria list for DILI induced by anti-TB treatment included (Global tuberculosis report 2022, 2022) a clear history of taking first-line anti-TB drugs, including isoniazid, rifampicin, ethambutol and pyrazinamide (Global tuberculosis report 2021, 2021). The peak values in liver function test within 180 days following the first drug dose met at least one of these followed terms alanine aminotransferase (ALT) ≥ 3 times the upper limit of normal range (ULN); total bilirubin ≥2 times ULN; under the premise of elevated levels of aspartate aminotransferase (AST), alkaline phosphatase and total bilirubin, at least one ≥2 times ULN. The alcohol consumption was defined as the patient had daily intake of alcohol based on self-description after admission.

### Screening the variables to establish a prediction model

**FIGURE 1:**
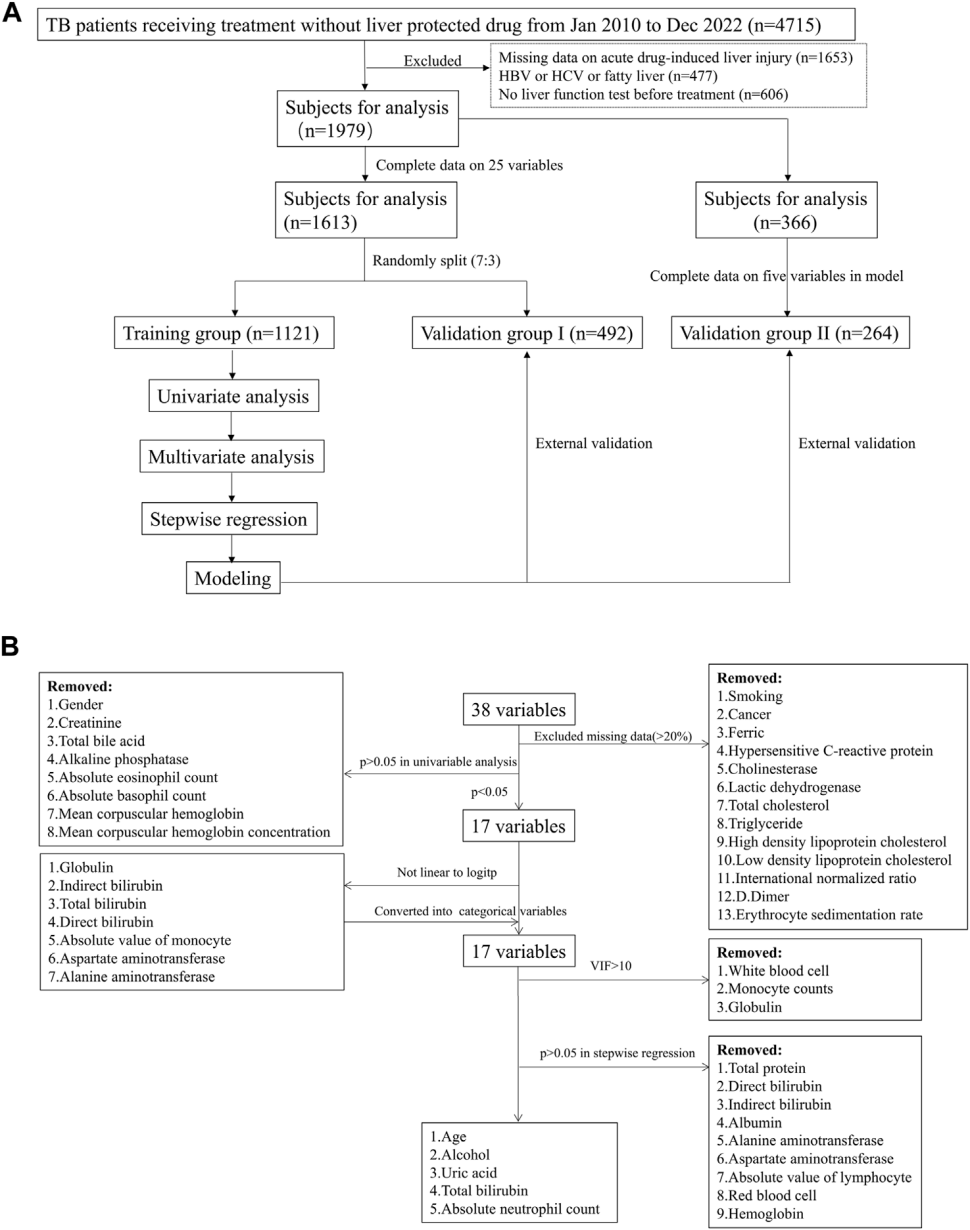
Study flowchart in the model establishment. **(A)** patient inclusion and exclusion in the model; **(B)** variables screening process in the model.

All the candidate variables contained clinical and laboratory indexes, which were the last measured values within 30 days before drug use. The variables with missing percentages higher 20% were removed, and the enrolled patients with complete data on the remaining variables were randomly split into training group and validation group I with a ratio of 7:3 (Figure 1A). The process for the identification of the variables used in generating the model was described in Figure 1B. First, univariate analysis was performed to identify variables significantly related to DILI (*p* < 0.05). The linear relationship between the continual variables and logitp was tested using the Box-Tidwell function. If, a linear relationship was absent (*p* < 0.05), and the variable was converted into categorical variables based on the normal range. The presence of multicollinearity among the involved variables was defined as variance inflation factors (VIFs) larger than 10, and thus these variables were excluded in further analysis. Second, multivariate analysis and stepwise regression were used to identify variables significantly related to DILI. Based on the finally involved variables, a second validation group (validation group II) was extracted from the population, excluding those had been enrolled in training group and validation group I. The discriminative ability of logistic regression model was assessed based on the area under the receiver operating characteristic (ROC) curve (AUC), model calibration was evaluated using the Hosmer–Lemeshow statistic (*p* > 0.05 indicated good agreement between predicted and real values) and the net benefit in clinical was evaluated by decision curve analysis (DCA). The prediction power of established model was further validated in two validation groups (Figure 1A). Finally, a risk-assessment model for DILI was developed and displayed as a nomogram graph in the training group.

### Statistical analysis

The SPSS (version 23) and R 4.2.1 software for Windows were used for statistical analysis. Continuous variables were expressed as median with the corresponding interquartile range, and were compared using either Student’s *t*-test or the Mann–Whitney *U* test. Categorical variables were compared using the χ^2^ test or Fisher’s exact test. A *p*-value <0.05 was considered of statistical significance**.**

## Results

### Characteristics of the study population

A total of 4,715 patients under anti-TB drug treatment were initially included in this study (Figure 1A). 2,259 patients were excluded due to missing information on liver function test in our hospital after receiving treatment (n = 1,653) or before the drug therapy (n = 606). There were 477 patients owned baseline liver diseases including hepatis B, hepatis C or fatty liver, then were removed in further analysis. In the enrolled 1979 patients, 83 (4.2%) developed DILI within 180 days after treatment. There were 25 variables included in analysis after removing the variables with missing data higher than 20% (Figure 1B). Then, 1,613 patients with complete variables were divided into training group (n = 1,121) and validation group I (n = 492), with no significant differences for the involved variables (Supplementary Table S1).

### Identification of the variables to be included in the model

Univariate analysis showed that 17 clinical or laboratory variables were significantly associated with DILI (*p* < 0.05, Table 1 and Figure 1B). Gender, creatinine, total bile acid, alkaline phosphatase, absolute eosinophil count, absolute basophil count, mean corpuscular hemoglobin and mean corpuscular hemoglobin concentration were not found significantly associated with DILI (*p* > 0.05). Seven variables were converted into categorical variables, including globulin, indirect bilirubin, total bilirubin, direct bilirubin, absolute value of monocyte, aspartate aminotransferase and alanine aminotransferase due to no linearity to logitp (Figure 1B). Three variables (white blood cell, monocyte counts and globulin) were excluded because of multi-collinearity. Among the remaining variables, there was no multi-collinearity (Supplementary Table S2).

**TABLE 1:** Univariable analysis of DILI related variables in the training group^a^.

Variable	Total	Non-DILI	DILI	*P* ^b^
	N = 1,121	N = 1,080	N = 41	
Gender, n (%)				0.118
Female	417 (37)	407 (38)	10 (24)	
Male	704 (63)	673 (62)	31 (76)	
Age	50 (29, 68)	49 (28.75, 68)	62 (52, 76)	<0.001
Alcohol consumption, n (%)				<0.001
No	961 (86)	936 (87)	25 (61)	
Yes	160 (14)	144 (13)	16 (39)	
Uric acid (μmol/L)	287 (222, 366)	290 (223, 369)	237 (159, 323)	<0.001
Creatinine (μmol/L)	61 (51, 71)	61 (51, 71)	68 (54, 73)	0.073
Total bilirubin (μmol/L)	9.1 (6.9, 12.4)	9 (6.9, 12.1)	17.5 (10.2, 20.8)	<0.001
Direct bilirubin (μmol/L)	3.4 (2.5, 4.7)	3.3 (2.5, 4.6)	6.2 (4.1, 9.8)	<0.001
Indirect bilirubin (μmol/L)	5.6 (4.2, 7.9)	5.5 (4.2, 7.8)	9 (6.1, 11.9)	<0.001
Total bile acid (μmol/L)	3.3 (2, 5.8)	3.3 (2, 5.8)	3.3 (2.1, 6.1)	0.588
Total Protein (g/L)	68.73 ± 7.92	68.94 ± 7.8	63.35 ± 9.28	<0.001
Albumin (g/L)	39 (34.2, 44)	39.2 (34.48, 44.1)	35 (31.7, 39.3)	<0.001
Globulin (g/L)	29.2 (25.9, 32.7)	29.3 (26, 32.7)	27.8 (23.5, 31.9)	0.03
Alanine aminotransferase (U/L)	15 (11, 23)	15 (11, 23)	18 (13, 30)	0.029
Aspartate aminotransferase (U/L)	20 (17, 26)	20 (16, 26)	25 (19, 31)	0.003
Alkaline phosphatase (U/L)	77 (62, 96)	77 (62.75, 96)	67 (60, 88)	0.257
White blood cell (x10^9/L)	6.29 (5.14, 7.65)	6.27 (5.11, 7.62)	7.37 (5.94, 9.7)	0.002
Neutrophil count (x10^9/L)	4.09 (3.14, 5.37)	4.05 (3.11, 5.32)	4.66 (4.15, 6.95)	<0.001
Lymphocyte count (x10^9/L)	1.43 (1.08, 1.82)	1.44 (1.09, 1.82)	1.18 (0.85, 1.6)	0.009
Monocyte counts (x10^9/L)	0.47 (0.36, 0.63)	0.47 (0.35, 0.62)	0.57 (0.45, 0.73)	0.001
Eosinophil count (x10^9/L)	0.12 (0.06, 0.19)	0.12 (0.06, 0.19)	0.09 (0.04, 0.17)	0.077
Basophil count (x10^9/L)	0.02 (0.01, 0.03)	0.02 (0.01, 0.03)	0.02 (0.01, 0.03)	0.789
Hemoglobin (g/L)	129 (114, 142)	129 (115, 142)	120 (101, 135)	0.015
Red blood cell (x10^12/L)	4.39 (3.94, 4.86)	4.4 (3.97, 4.86)	3.89 (3.52, 4.58)	<0.001
Mean corpuscular hemoglobin (pg)	29.5 (28.3, 30.7)	29.5 (28.3, 30.7)	29.9 (28.7, 31.6)	0.101
Mean corpuscular hemoglobin concentration (g/L)	331 (323, 339)	331 (323, 338)	333 (324, 339)	0.46

^a^ Continual variables were displayed as Median (IQR), categorical variables were displayed as number (percentage).

^b^ Categorical variables were compared by Pearson’s Chi-squared test, continual variables were analyzed by Wilcoxon rank sum test.

Finally, five parameters (age, alcohol, uric acid, total bilirubin and neutrophil count) were significantly related to DILI by stepwise regression (Table 2; Figure 1B). According to odds ratio (OR), the following four parameters were high-risk factors for DILI in patients with TB during anti-TB treatments: age (OR 1.022 [1.003–1.042], *p* = 0.023), elevated total bilirubin (OR 11.714 [5.837–23.692], *p* < 0.001), neutrophil count (OR 2.145 [1.078–4.700], *p* = 0.013), and alcohol consumption (OR 3.209 [1.520–6.652], *p* = 0.002). Conversely, uric acid (OR 0.977 [0.993–1.000], *p* = 0.047) was the protective parameter against the development of DILI (Table 2).

**TABLE 2:** The multiple logistic regression analysis of risk factors for LIDI in the training group.

Variables	Logistic regression	
	Or (95% CI)	*P*
Age	1.022 (1.003–1.042)	0.023
Alcohol consumption	3.209 (1.520–6.652)	0.002
Uric acid (μmol/L)	0.977 (0.993–1.000)	0.047
Elevated total bilirubin (≥17.1 μmol/L)	11.714 (5.837–23.692)	<0.001
Neutrophil count (10^9/L)	2.145 (1.078–4.700)	0.013

### Development of a risk-prediction model for drug-induced liver injury in the training group

**FIGURE 2:**
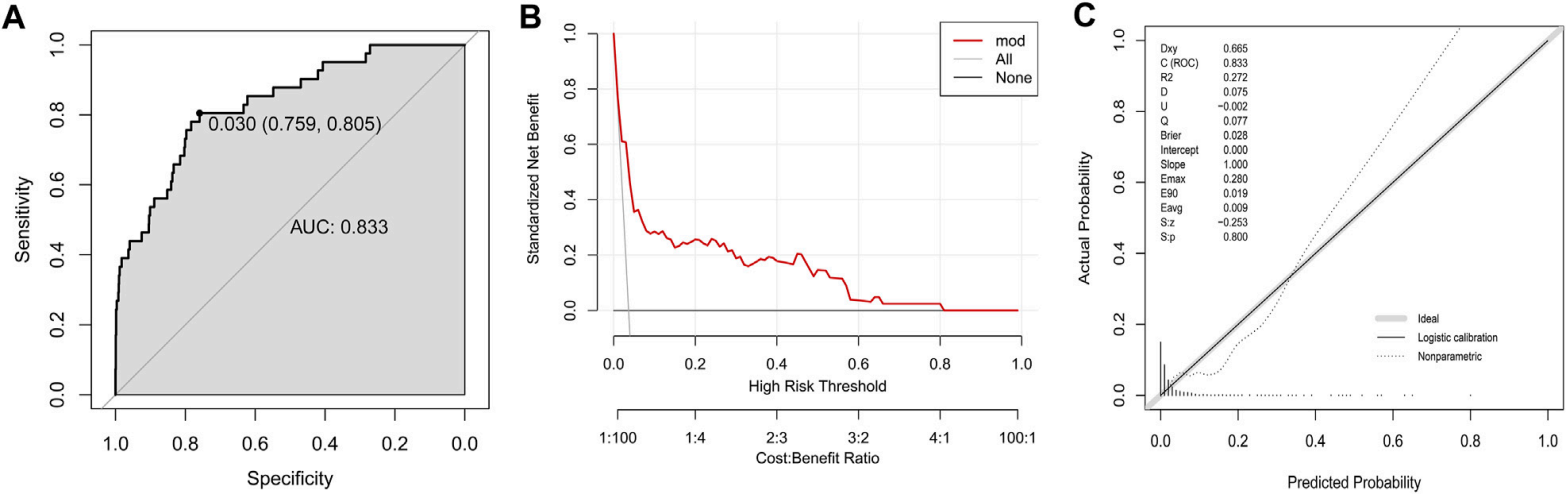
Development of a model to predict DILI in the training group. **(A)** The ROC curve; **(B)** Decision curve; **(C)** Calibration plot.

To evaluate the discriminative ability of the logistic regression model, the final prediction model was constructed in the training group based on five variables: age, elevated total bilirubin, neutrophil count, uric acid and alcohol consumption. The AUC of prediction model was 0.833, with a sensitivity of 0.759 and specificity of 0.805 at the threshold of 0.030 (Figure 2A). The DCA curve of established model was far from the two reference curves (Figure 2B). The calibration curve owned a *p*-value of 0.800 based on the Hosmer & Lemeshow test (Figure 2C).

### Validation of the risk-prediction model in external data sets

**FIGURE 3:**
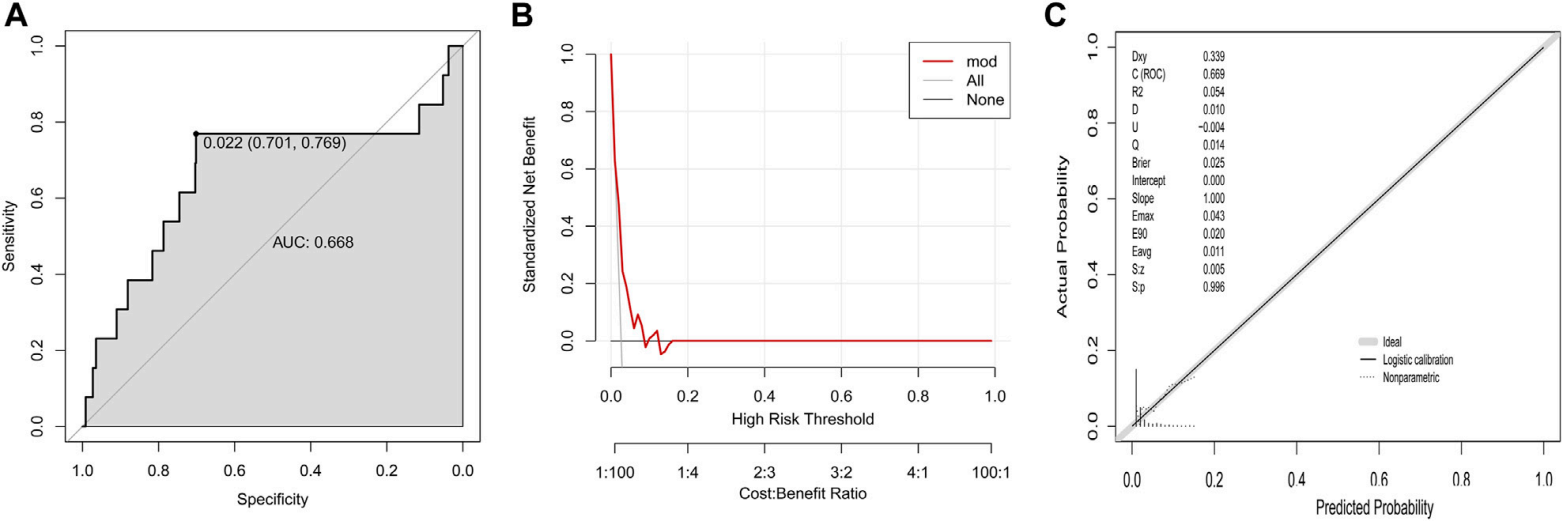
Validation of established model to predict DILI risk in the validation group I **(A)** ROC curve; **(B)** Decision curve; **(C)** Calibration plot.

In the validation group I that owned comparable baseline variables to training group, this established model showed a moderate discrimination power (AUC, 0.668, Figure 3A). The DCA curve in the validation group I was near to the two reference lines (Figure 3B). For calibration curve, the *p*-value based on Hosmer & Lemeshow test was 0.996 (Figure 3C).

**FIGURE 4:**
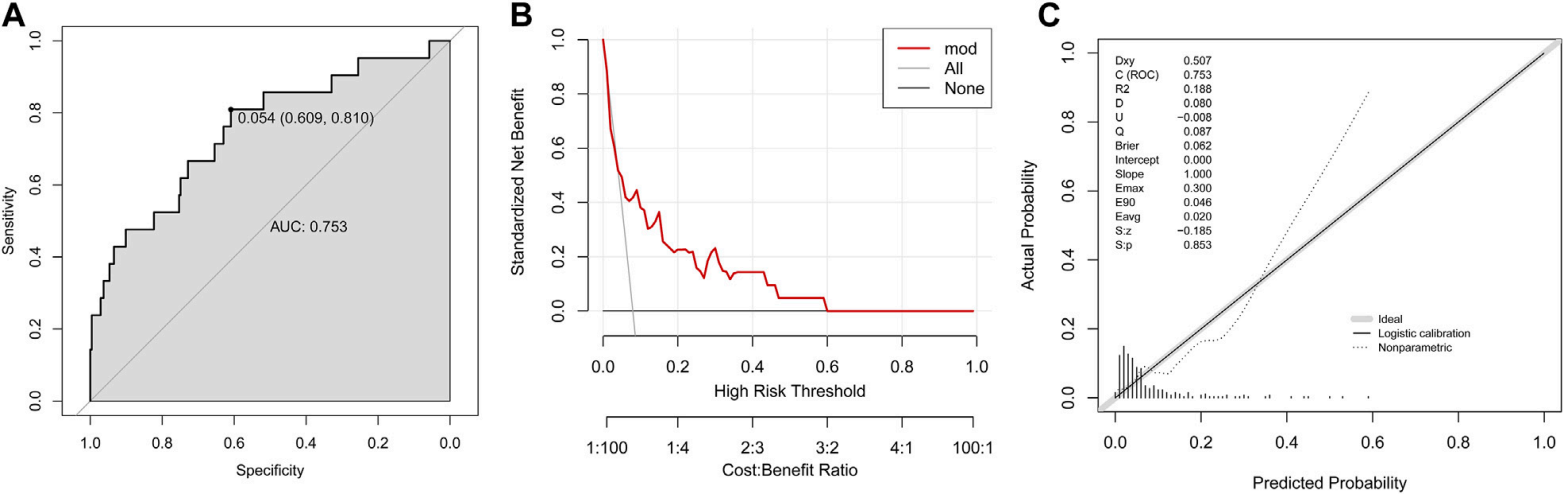
Validation of established model to predict DILI risk in the validation group II. **(A)** ROC curve; **(B)** Decision curve; **(C)** Calibration plot.

Another validation population (validation group II, n = 264) was extracted from the 1979 patients with complete information on final five variables after excluding those involved in training group and validation group I. Fourteen variables showed significant differences between training group and validation group II (Supplementary Table S1). The established prediction model in validation group II owned an AUC of 0.753 (Figure 4A), a DCA curve far from the two reference lines (Figure 4B) and a *p*-value of 0.853 in calibration curve (Figure 4C).

### Establishment of a nomogram graph

**FIGURE 5:**
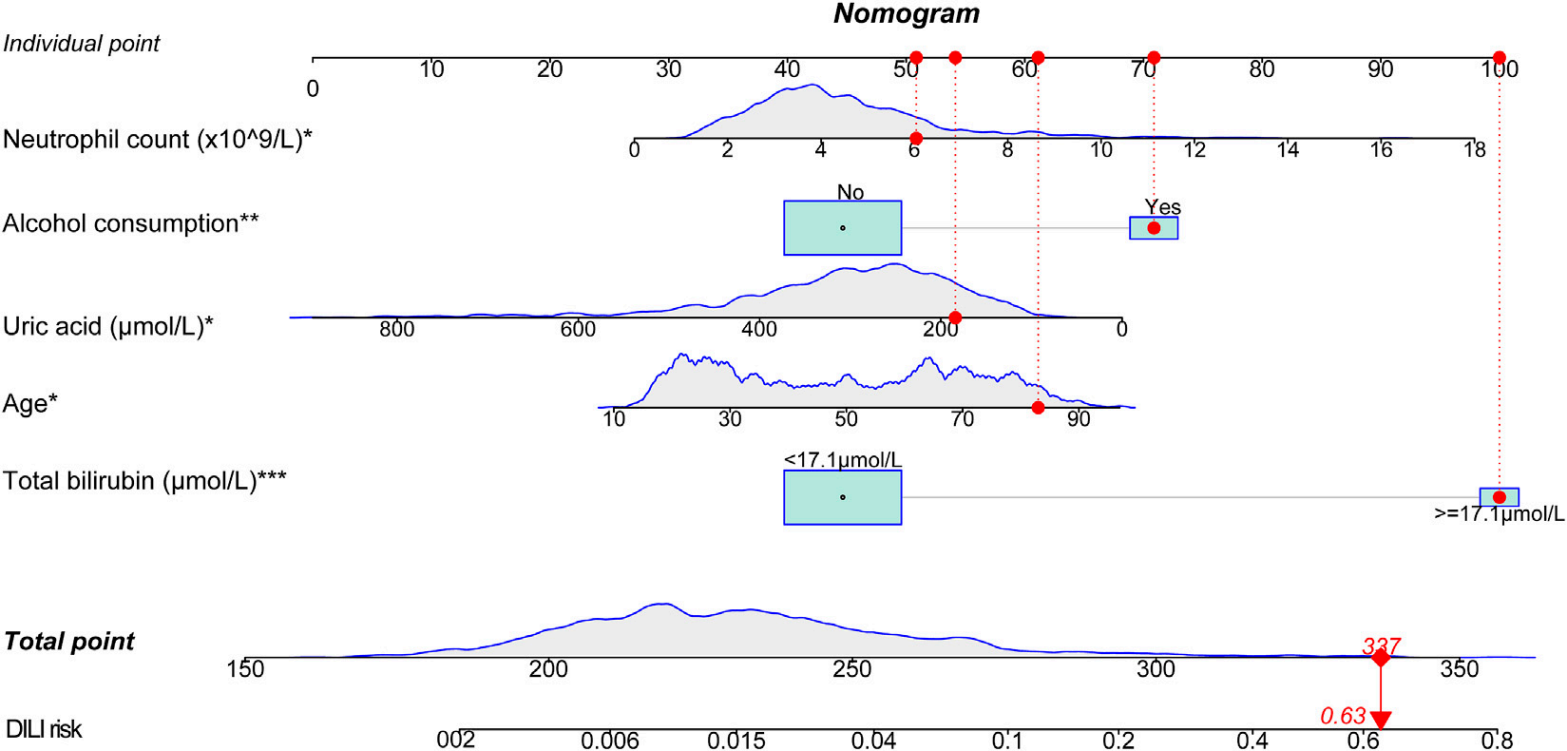
Nomogram for predicting DILI in the training group. A patient was given as an example indicated by red dots in figure (81 years old, total bilirubin of 52 μmol/L, uric acid of 159 μmol/L, neutrophil count of 6.41 × 10^9/L, with alcohol consumption). The total score is 337 points, corresponding to a DILI risk of 0.63. The variables with symbol * indicated for significance in the model, *p < 0.05, **p < 0.01, ***p < 0.001.

Based on the established logistic regression model, a nomogram for presenting the significance of involved variables for predicting the risk of DILI among the patients was established (Figure 5). Individual points corresponding to each measured variable could be obtained by vertically matching them to the top reference point line, and total point could be calculated by summing all the individual points. The risk of DILI could be read by vertically matching the total point to the risk line.

## Discussion

DILI in patients receiving anti-TB agents can interrupt the therapy process, and identification of associated risk factors can help clinical staff to take preventive measures against it. Here, a nomogram model was established based on five clinical parameters (age and levels of total bilirubin, uric acid, neutrophil account, and alcohol consumption) assessed before the treatment onset. This model could predict the DILI risk in patients receiving first-line anti-TB agents who did not take any hepatoprotectants, and has a good discrimination power.

A 6-month regimen is recommended by WHO composing of four first-line anti-TB agents (isoniazid, rifampicin, ethambutol, and pyrazinamide) for patients with TB (W HO, 2022). The regimen could be modified based on the age of the patient, resulting in a treatment success rate >85%. However, DILI is a serious side effect of such regimens, with an incidence rate of 2.0%–28%, and may largely be due to the pyrazinamide in the regimen (Ghosh et al., 2021). Among the patients analyzed in this study, 4.3% suffered from DILI. The incidence of hepatotoxicity in this study might be lower than that reported by others, as patients with baseline liver diseases and receiving liver protective drugs were excluded (Abbara et al., 2017; Latief et al., 2017; Raj Mani et al., 2021). This study assessed several possible factors for their ability to predict future DILI among TB patients negative for liver-related diseases, who might be easily ignored by doctors. Moreover, hepatoprotectants were not used in enrolled patients, giving the chance to investigate the real DILI incidence and related variables (Chen et al., 2022).

In this study, five objective parameters were involved in the prediction model, and these parameters can be easily obtained in clinical practice before the anti-tuberculosis treatment. Elevated levels of total bilirubin might be associated with metabolism dysfunction in liver, eventually increasing the DILI risk once the treatment starts. As the sum of indirect bilirubin and direct bilirubin, the increased total bilirubin might be the results of reduced uptake ability by the liver and weakened excretion by the kidney. In this study, the kidney related variable creatinine was comparable between patients with DILI and without DILI, indicating for key role of liver dysfunction in elevated total bilirubin instead of kidney impairment. Though other studies have indicated the significance of ALT and AST levels for predicting DILI, these two variables were not the significant factors in this study (Mushiroda et al., 2016; Jiang et al., 2021). The inconsistency could be partly due to the differences in patients’ inclusion criteria, as patients with baseline liver diseases owned higher DILI risks (Jiang et al., 2021). In this study, uric acid was negatively associated with DILI risk, which could be partly explained by its anti-oxidant role in plasma (El Ridi and Tallima, 2017). In another way, lower uric acid in plasma might be caused by impaired reabsorption by kidney, with consequently increased uric acid in urine from patients with DILI revealed by urine metabolomics (Wang et al., 2022b). Moreover, neutrophil was positively associated with the risk of DILI in this study, which is consistent to the fact that neutrophil is always involved in inflammation and excessive or dysfunctional neutrophils lead into tissue damage (Wang and Liu, 2021). The high level of neutrophil in blood would provide enough source for infiltrating into liver when accumulative cytotoxic drug metabolites impaired the hepatocytes, resulting into an increased risk of liver injury (Cho and Szabo, 2021). Alcohol consumption and age were also found closely associated with DILI in this study, and these two factors have been widely reported by others (Abbara et al., 2017; Yew et al., 2018; Song et al., 2019).

Although several studies on DILI risk factors have been conducted, these studies involved limited numbers of patients and mostly focused on screening for risk factors (Lee et al., 2016; Medina-Caliz et al., 2016; Mushiroda et al., 2016; Raj Mani et al., 2021). Our nomogram prediction model could show the exact risk based on five parameters and has a good predictive ability regarding the ROC value, sensitivity, and specificity (Zhao et al., 2022). By using the XGBoost algorithm, Zhong et al. have established a model that can predict DILI with high accuracy and interpretability. However, their model involves the accumulative dose of anti-TB agents used during a treatment course, and this dose cannot be foreseen before starting the treatment (Zhong et al., 2021). In the validation step, two groups were enrolled in this study, representing for populations with comparable baselines and with different baselines, respectively. The prediction ability of established model in training model reduced in validate group with comparable baselines, which might be caused by limited DILI cases in validation. When validated in group with different baselines, the model showed increased prediction ability comparing to group with comparable baselines, suggesting its application possibility in other population.

There were some limitations in this study. Despite the large sample size, all the patients were from a single center, and thus the prediction power of established model need be validated in other regions. The duration between the treatment onset and DILI occurrence was not available from the database. Several parameters were not available for some of the involved patients due to the retrospective nature of the study, resulting in the exclusion of more than 50% of the patients. In addition, the treatment duration and accumulative drug dosage were not available from the database.

## Conclusion

TB patients frequently suffer from DILI after receiving first-line anti-TB agents, though they were not accompanied by liver-related diseases before treatment, including HBV infection, fatty liver and liver cancers. A nomogram model based on five variables before anti-TB treatment (age, total bilirubin, neutrophil count, alcohol consumption and uric acid) can predict future DILI with a good discriminative power. This prediction model based on these easily accessible parameters can help clinical staff to evaluate the DILI risk before the treatment and take active intervenes to prevent the occurrence of DILI in patients with high risk.

## Data Availability

The original contributions presented in the study are included in the article/Supplementary Material, further inquiries can be directed to the corresponding author.
