# Supplementary material for: A nomogram model to predict the risk of drug-induced liver injury in patients receiving anti-tuberculosis treatment
Source: Front Pharmacol. 2023 May 18;14:1153815. doi: 10.3389/fphar.2023.1153815 (PMC10232814; doi:10.3389/fphar.2023.1153815)
Supplement: Supplementary file 1 [file DataSheet1.docx]

Supplementary table 1. Baseline characteristics in the training and validation groups ^a^.

| Variable | Training group,  N = 1211 | Validation group I  N = 492 | *P* ^b^ | Validation group II  N = 264 | *P* ^b^ |
| --- | --- | --- | --- | --- | --- |
| Gender, n (%) |  |  | 0.427 |  | 0.449 |
| Female | 417 (37) | 194 (39) |  | 91 (34) |  |
| Male | 704 (63) | 298 (61) |  | 173 (66) |  |
| Age | 50 (29, 68) | 52 (31, 69) | 0.232 | 66 (42.75, 77) | < 0.001 |
| Alcohol consumption, n (%) |  |  | 0.353 |  | 0.098 |
| No | 961 (86) | 431 (88) |  | 215 (81) |  |
| Yes | 160 (14) | 61 (12) |  | 49 (19) |  |
| Uric acid (μmol/L) | 287 (222, 366) | 291 (233, 367) | 0.344 | 244 (183.5, 304.75) | < 0.001 |
| Creatinine (μmol/L) | 61 (51, 71) | 60.5 (52, 71.25) | 0.613 | 62 (48.25, 73) | 0.825 |
| Total bilirubin (μmol/L) | 9.1 (6.9, 12.4) | 9.4 (6.97, 13.03) | 0.188 | 9.3 (7.3, 13.03) | 0.264 |
| Direct bilirubin (μmol/L) | 3.4 (2.5, 4.7) | 3.4 (2.5, 5.1) | 0.564 | 3.85 (2.7, 5.35) | < 0.001 |
| Indirect bilirubin (μmol/L) | 5.6 (4.2, 7.9) | 5.95 (4.3, 8.2) | 0.101 | 5.6 (4.1, 7.3) | 0.548 |
| Total bile acid (μmol/L) | 3.3 (2, 5.8) | 3.3 (2, 5.5) | 0.709 | 4.6 (2.3, 7.9) | < 0.001 |
| Total Protein (g/L) | 68.9 (63.2, 74.4) | 68.8 (62.9, 74.7) | 0.907 | 62.85 (57.82, 69.65) | < 0.001 |
| Albumin (g/L) | 39 (34.2, 44) | 39.2 (34.18, 43.6) | 0.961 | 34.8 (30.5, 39) | < 0.001 |
| Globulin (g/L) | 29.2 (25.9, 32.7) | 29.4 (25.87, 32.92) | 0.836 | 28.2 (24.4, 31.8) | 0.002 |
| Alanine aminotransferase (U/L) | 15 (11, 23) | 14 (10, 22) | 0.07 | 16 (10, 24) | 0.536 |
| Aspartate aminotransferase (U/L) | 20 (17, 26) | 21 (17, 25) | 0.813 | 21 (16, 28) | 0.774 |
| Alkaline phosphatase (U/L) | 77 (62, 96) | 78 (64, 95) | 0.61 | 75.5 (61.75, 94) | 0.414 |
| White blood cell (x10^9/L) | 6.29 (5.14, 7.65) | 6.3 (5.13, 7.54) | 0.665 | 6.58 (4.73, 9.45) | 0.052 |
| Absolute neutrophil count (x10^9/L) | 4.09 (3.14, 5.37) | 4.04 (3.19, 5.33) | 0.887 | 5.02 (3.24, 7.36) | < 0.001 |
| ^a^ Continual variables were displayed as Median (IQR), categorical variables were displayed as number (percentage); | | | | | |
| ^b^ Categorical variables were compared to the training group by Pearson's Chi-squared test, continual variables were analyzed by Wilcoxon rank sum test. | | | | | |

Supplementary table 2. The linearity test between the involved continual variables and logitp by boxTidwell test.

| Variable | Statistic | *p* value |
| --- | --- | --- |
| Age | 1.1059 | 0.2688 |
| Uric acid (μmol/L) | 0.7982 | 0.4248 |
| Total Protein (g/L) | 0.4608 | 0.6449 |
| Albumin (g/L) | -1.2089 | 0.2267 |
| White blood cell (x10^9/L) | -0.5112 | 0.6092 |
| Absolute neutrophil count (x10^9/L) | 0.6167 | 0.5374 |
| Absolute value of lymphocyte (x10^9/L) | 0.8429 | 0.3993 |
| Hemoglobin (g/L) | 0.6982 | 0.485 |
